# Modulation of oxazolone-induced hypersensitivity in mice by selective PDE inhibitors

**DOI:** 10.1155/S0962935195000196

**Published:** 1995-03

**Authors:** I. Moodley, Y. Sotsios, B. Bertin

**Affiliations:** 1 Institut de Recherche Jouveinal 9 rue de la Loge Fresnes Cedex 94265 France; 2 Department of Pharmacy University of the Witwatersrand Medical School Parktown Johannesburg 2193 South Africa

## Abstract

The effects of PDE inhibitors on oxazolone-induced contact hypersensitivity (CS) were studied in mice. Rolipram, Ro 20-1724 and theophylline dose dependently inhibited CS but none caused >53% inhibition. ED_30_ values at 24 h before challenge for rolipram, Ro 20-1724 and theophylline were 2.1, 5.4 and 30.4 mg/kg, p.o., respectively. Milrinone and SKF 94836 at 30 mg/kg caused a small, but significant inhibition of 13% and 18%, respectively, although the inhibition (8%) caused by zaprinast was not significant. Betamethasone (10 mg/kg, p.o.) caused a marked inhibition (80%) as did indomethacin (65% at 5 mg/kg, p.o.). Rolipram and Ro 20-1724 inhibited proliferation of mouse lymphoblasts with IC_50_ values of 0.08 μM and 0.83 μM, respectively. In contrast, zaprinast caused only a weak inhibition (IC_50_ = 119 μM) of lymphocyte proliferation, whereas SKF 94836 and theophylline failed to cause any significant inhibition at 100 μM (26% and 2%, respectively). These findings suggest that PDE IV isozymes play a principal role in mediating CS by inhibiting lymphocyte activation.


Mediators of Inflammation 4, 112–116 (1995)

### Modulation of oxazolone-induced hypersensitivity in mice by selective PDE inhibitors

I. Moodley,* Y. Sotsios and B. Bertin[CA]

Institut de Recherche Jouveinal, 9 rue de la Loge, 94265 Fresnes Cedex, France
*Present address: Department of Pharmacy, University of the Witwatersrand Medical School, Parktown, 2193, Johannesburg, South Africa

[CA] Corresponding Author

**The effects of PDE inhibitors on oxazolone-induced contact hypersensitivity (CS) were studied in mice. Rolipram, Ro 20-1724 and theophylline dose dependently inhibited CS but none caused >53% inhibition. $ED_{30}$ values at 24 h before challenge for rolipram, Ro 20-1724 and theophylline were 2.1, 5.4 and 30.4 mg/kg, p.o., respectively. Milrinone and SKF 94836 at 30 mg/kg caused a small, but significant inhibition of 13% and 18%, respectively, although the inhibition (8%) caused by zaprinast was not significant. Betamethasone (10 mg/kg, p.o.) caused a marked inhibition (80%) as did indomethacin (65% at 5 mg/kg, p.o.). Rolipram and Ro 20-1724 inhibited proliferation of mouse lymphoblasts with $IC_{50}$ values of 0.08 μM and 0.83 μM, respectively. In contrast, zaprinast caused only a weak inhibition ($IC_{50}$ = 119 μM) of lymphocyte proliferation, whereas SKF 94836 and theophylline failed to cause any significant inhibition at 100 μM (26% and 2%, respectively). These findings suggest that PDE IV isozymes play a principal role in mediating CS by inhibiting lymphocyte activation.**

**Key words**: Contact hypersensitivity, Oxazolone, PDE inhibitors

### Introduction

In inflammatory diseases such as allergic or contact dermatitis, there is convincing evidence for the involvement of activated leukocytes, such as lymphocytes, eosinophils and monocytes.[1] The regulation of the activation state of these leukocytes occurs at many levels. Evidence is now accumulating that changes in the intracellular levels of cyclic nucleotides, such as cyclic adenosine monophosphate (cAMP) play an important role in the regulation of inflammatory cell activity[2,3] and that in atopic dermatitis there is decreased cyclic nucleotide phosphodiesterase (PDE) activity.[4] In general, increases in levels of cAMP result in a reduction in the activity of inflammatory cells. The rate of breakdown of cAMP is controlled by a family of five isozymes collectively known as cyclic nucleotide phosphodiesterases and are designated PDE I to V.[5] These isozymes are classified according to their differences in tissue distribution, kinetic and physical characteristics, substrate selectivities, sensitivities to endogenous activators and inhibitors, susceptibility to phosphorylation by protein kinases and protein sequences. PDE IV isozymes appear to a very large extent to be present in inflammatory cells, such as basophils, mast cells, neutrophils, monocytes and eosinophils. In contrast there appears to be both PDE III and PDE IV isozymes present in lymphocytes.[2,6,7]

Cluster differentiation (CD) analysis has permitted the classification of lymphocytes according to their different functions.[8] Helper, $CD4^+$ lymphocytes are involved in immediate hypersensitivity and both $CD4^+$ and $CD8^+$ lymphocytes are involved in delayed or contact type hypersensitivity.[9] $CD4^+$ lymphocytes have been differentiated into Th1 and Th2 subtypes according to their lymphokine production, at least in the mouse. However, functional differentiation of lymphocytes has also been demonstrated in patients with infections and allergies.[10]

The purpose of this study was to investigate to what extent selective PDE inhibitors, milrinone and SKF 94836 (PDE III), Rolipram and Ro 20-1724 (PDE IV), zaprinast (PDE V) and the non-selective PDE inhibitor, theophylline, were capable of modulating oxazalone-induced ear oedema in the mouse, an animal model of contact hypersensitivity. Such a study could thus provide a tentative insight into the relative importance of PDE isozymes in modulating the activity of subsets of lymphocytes that mediate the contact hypersensitivity response.

### Materials and Methods

*Mice:* Male Swiss mice (16–18 g on arrival) and female, C57B16 (5–6 weeks old) were used for the contact hypersensitivity and lymphoblast transformation experiments, respectively, and were purchased from IFFA CREDO, France. The animals were acclimatized for a period of 4 –7 days prior to use, at an ambient temperature of 21°C under artificial light

© 1995 Rapid Communications of Oxford Ltd with a 12 h cycle. Food (with added vitamins, A114, UAR) and water was available *ad libitum*.

*Contact hypersensitivity:* Mice were sensitized by application of 0.1 ml of 2% (w/v) oxazolone (4-ethoxymethylene-2-phenyl-2-oxazol-5-one, Sigma Chemicals, MO, USA) in olive oil to the shaved abdominal area. Seven days later mice were challenged with oxazolone 2% (w/v), in a vehicle consisting of 2-methoxyethanol:95% ethanol:Tween 80 (45:45:10), applied externally on the inside surface of the right ear, and vehicle alone to the left ear. Volumes of administration were 10 μl/ear.

*Measurements:* Ear thickness was measured at 6 h and 24 h after challenge. Both ears were measured using a micrometer (Digitrix II, Helios W5126001) and the difference in volume between the two ears were noted for further analysis. Animals were anaesthetized with pentobarbital for the 6 h measurement and sacrificed by cervical dislocation for the 24 h measurement.

*Treatment:* PDE inhibitors or glucorticosteroids were given orally as a single administration either at 24 h or 3 h before challenge or 3 h after challenge.

*Statistical evaluation:* Results were analysed statistically using the unpaired Student's *t*-test. In each treatment group there were ten mice. Values are means of three separate experiments.

*Lymphoblast transformation:* Mice were sacrificed by decapitation. Thymus glands were removed and placed in Spinner modified minimal essential medium (SMEM) supplemented with 1% v/v non-essential amino acids, 1.2 mM L-glutamate, 1 mM sodium pyruvate, 100 U/ml penicillin, 100 μg/ml streptomycin and 5% Ultroser, cleaned of adherent connective tissue and placed on a 40 micron mesh nylon gauze and dispersed with the aid of a piston from a 5 ml syringe. The cell suspension was then centrifuged at 250 × **g** for 10 min at 4°C and resuspended in SMEM. The viability of the cells assessed by Trypan Blue exclusion were usually >90%. Aliquots containing $1 \times 10^6$ cells were distributed into 96-well flat-bottomed microtitre plates (Falcon) and incubated for 24 h at 37°C in a humidified atmosphere of 95% air and 5% $CO_2$ in the presence of concanavalin A (5 μg/ml) and appropriate concentrations of the different types of PDE inhibitors dissolved in dimethyl sulphoxide which had a final concentration of 0.25% v/v in the incubation medium and did not affect the proliferative response. Following the addition of [$^3$H]-thymidine (0.1 μCi), the cells were then incubated for a further 24 h. Cells were then collected onto glass fibre filters (GF/A, Whatman) with the aid of a PHD Cell Harvester and placed into scintillation vials containing Scintillator Plus (Packard) for counting in Betamatic Scintillation Counter (Kontron) for 1 min.

*Statistical evaluation:* Each PDE inhibitor was examined in triplicate on thymus cell preparations from three mice. Percentage inhibition was calculated with respect to vehicle treated controls and $IC_{50}$ values determined where applicable and analysed statistically using Dunnett's test.

*Materials:* Theophylline, milrinone and indomethacin were purchased from Sigma Chemicals, MO, USA; pentobarbital from Sanofi, France. Ro 20-1724 was from RBI. SKF 94836 and zaprinast were generous gifts from Smith Kline & Beecham and Rhone–Poulenc–Rorer, UK. Rolipram, prednisolone and betamethasone were synthesized at Institut de Recherche Jouveinal.

SMEM, non-essential amino acids, L-glutamate, sodium pyruvate, penicillin and streptomycin were purchased from Gibco, France. Ultroser and [$^3$H]-thymidine were purchased from Sepracor, France and NEN, Belgium, respectively.

### Results

*Effect of PDE inhibitors on the inhibition of oxazolone-induced ear-swelling in mice, measured at 6 h or 24 h after challenge:* The selective PDE IV inhibitors, rolipram and Ro 20-1724 as well as the non-selective PDE inhibitor, theophylline, administered 24 h before challenge caused a dose-dependent inhibition of oxazolone-induced ear swelling measured either at 6 h or 24 h after challenge (Table 1). The effects were more pronounced when ear swelling was measured 24 h after challenge. However, even at maximal doses, these PDE inhibitors failed to cause more than 53% inhibition. Indomethacin also caused a dose-related inhibition that did not appear to be dependent on the time of measurement. Furthermore, at the highest dose of indomethacin, inhibition of ear swelling was 66% and 60%, measured either at 6 h or 24 h after challenge. Thus for theophylline and the selective PDE IV inhibitors and indomethacin $ED_{30}$ values (i.e., the dose in mg/kg resulting in 30% inhibition) were determined as shown in Table 2. The order of potency was indomethacin > rolipram > Ro20-1724> theophylline, the activity of the PDE inhibitors being greater when measured 24 h after challenge as mentioned above.

*Effect of time of administration of selective PDE inhibitors on oxazolone-induced ear-swelling in mice, measured 24 h after challenge:* For these experiments all measurements of ear swelling were made 24 h after challenge and the test compounds were administered at 3 h or 24 h before challenge or 3 h after challenge. The results are shown in Table 3. Theophylline appeared to be more active when administered either 24 h or 3 h before challenge. The

**Table 1.** Effect of selective PDE inhibitors on inhibition of oxazolone-induced ear-swelling in mice, measured at 6 h or 24 h after challenge

| Compounds | Dose (mg/kg, p.o.) | Inhibition (%) | |
|---|---|---|---|
| | | 6 h after challenge | 24 h after challenge |
| challenge | | | |
| | 10 | -5.8 (ns) | 12.8 (ns) |
| Theophylline | 30 | 11.1 (ns) | 33.0*** |
| | 100 | 39.9*** | 45.2*** |
| | 1 | 10.6 (ns) | 20.2** |
| Rolipram | 3 | 27.5** | 35.4*** |
| | 5 | 38.6*** | 40.8*** |
| | 3 | 14.1 (ns) | 24.3*** |
| Ro 20-1724 | 10 | 24.5** | 34.5*** |
| | 30 | 37.2*** | 53.1*** |
| | 0.5 | 2.3 (ns) | 13.7* |
| Indomethacin | 1 | 24.1* | 33.9*** |
| | 3 | 33.2*** | 39.7*** |
| | 5 | 65.5*** | 59.3*** |
| Prednisolone | 5 | 25.4** | 38.3*** |

Compounds administered 24 h before challenge. $n = 3$, each group consisted of ten mice.
*$p < 0.05$, **$p < 0.01$; ***$p < 0.001$. ns, not significant.

**Table 2.** $ED_{30}$ values for selective PDE inhibitors administered 24 h before challenge

| Compounds | $ED_{30}$ values | |
|---|---|---|
| | 6 h after challenge | 24 h after challenge |
| Rolipram | 3.2 | 2.1 |
| Ro 20-1724 | 15.4 | 5.4 |
| Theophylline | nd | 30.4 |
| Indomethacin | 1.6 | 1.1 |

nd = not done.

activity Ro 20-1724 was similar regardless of times of administration. In contrast, rolipram appeared to be more active when administered either 24 h before or 3 h after challenge. The selective PDE III inhibitors, milrinone and SK&F 94836 and the PDE V inhibitors, zaprinast caused a smaller, but significant, inhibition and appeared to be more active when administered 3 h after challenge. For purposes of comparison, the glucocorticoid, betamethasone was also studied and caused a marked inhibition (77%) when administered 3 h before challenge. These differences in activity in relation to time of administration may reflect differences in metabolism or modulation of the activity of inflammatory cells at different levels of activation states following challenge.

*Effect of PDE inhibitors on concanavalin A induced proliferation of mouse lymphoblasts:* The effects of selective PDE inhibitors were studied on mitogen-induced thymocyte proliferation as a model of T cell activation. The results are shown in Table 4. Rolipram and Ro 20-1724 inhibited proliferation of mouse lymphoblasts with $IC_{50}$ values of 0.08 μM and 0.83 μM, respectively. In contrast, zaprinast caused only a weak inhibition ($IC_{50}$ = 119 μM), whereas SKF 94836 and theophylline failed to cause any significant inhibition at 100 μM (26% and 2%, respectively).

**Table 3.** Effect of selective PDE inhibitors following different times of administration on oxazolone-induced ear-swelling in mice, measured 24 h after challenge

| Compounds | Dose (mg/kg, p.o.) | Inhibition(%) | | |
|---|---|---|---|---|
| | | 24 h before challenge | 3 h before challenge | 3 h after challenge |
| Rolipram | 5 | 34.2*** | 27.0*** | 42.5*** |
| Ro 20-1724 | 30 | 47.5*** | 45.4*** | 45.2*** |
| Theophylline | 100 | 51.5*** | 52.9*** | 33.0*** |
| SKF 94836 | 30 | 13.4* | 13.4* | 22.2*** |
| Milrinone | 30 | 19.4** | 19.8** | 29.5*** |
| Zaprinast | 30 | 8.1 (ns) | 13.9* | 21.7** |
| Betamethasone | 10 | 20.7** | 76.7*** | nd |

$n = 3$, each group consisted of ten mice.
nd = not determined.
ns = $p > 0.05$, *$p < 0.05$, **$p < 0.01$, ***$p < 0.001$.

**Table 4.** Effect of selective PDE inhibitors on concanavalin A-induced proliferation of mouse thymocytes.

| Compounds | $IC_{50}$ (µM) Mean ± S.D. | *n* |
|---|---|---|
| Rolipram | 0.08 ± 0.06 | 10 |
| Ro 20-1724 | 0.83 ± 0.49 | 5 |
| Milrinone | 3.96 | 1 |
| SKF 94836 | >>100 | 2 |
| Zaprinast | 119.3 ± 53.7 | 5 |

*n* = number of experiments.

### Discussion

Cutaneous hypersensitivity responses with delayed kinetics are known as delayed type hypersensitivity (DTH) and contact sensitivity (CS) responses. These two types of hypersensitivity responses are characterized by the presence of lymphocytes and monocytes at the site of sensitivity.[11,12] In this work the effects of selective PDE inhibitors on oxazolone-induced contact hypersensitivity were studied in the mouse.

To our knowledge the PDE isozyme profile in mouse lymphocytes has not been studied. However, the PDE isozyme profiles of lymphocytes from rats[2,6] and humans[7] have been characterized. In rat lymphocytes types I, II, III, IV and V PDE isozymes have been identified. In human lymphocytes only types III and IV have been identified through partial characterization. In addition, in human T-cell lines, a rolipram insensitive, specific cAMP PDE has been identified. In terms of activation of lymphocytes by T-cell specific mitogens, PDE IV and to a lesser extent PDE III inhibitors attenuate the proliferative response through elevation of intracellular cAMP. Elevation of intracellular cAMP appears to differentially regulate the activity of Th1 and Th2. Forskolin or cholera toxin, agents that cause an increase in intracellular cAMP, inhibit proliferation and generation of IL-2 in Th1 cell lines but not proliferation and generation of IL-4 in Th2 cell lines.[13] However, elevation of cAMP through PDE inhibitors blocks the proliferative response in both Th1 and Th2 cell lines.[3] Thus it appears that elevation of intracellular cAMP at one level may be occurring in discrete pools and thus be able to control the activation state of different subsets of lymphocytes in a relatively precise manner. The response of different subsets of lymphocytes following sensitization with oxazolone has been studied. Three days after sensitization, $CD4^+$ and $CD8^+$ lymphocytes were isolated from draining lymph nodes and their lymphokine production assessed. At this stage $CD4^+$ Th1 lymphocytes appear to be important since IL-4 is necessary for transfer.

Our results on concanavalin A-induced proliferation of mouse lymphoblasts suggest that with the aid of selective PDE inhibitors the principal isozymes involved belong to the PDE IV subclass. As in the case for rat[2] and human[7] lymphocytes, PDE III isozymes also appear to contribute to some extent to the inhibition of mitogen-stimulated proliferative responses. In a manner similar to the effects of lymphocyte proliferation, we found that the selective PDE IV inhibitors, rolipram and Ro 20-1724 were the most potent inhibitors of oxazolone-induced hypersensitivity. However, this inhibitory effect was never greater than 50% and certainly much less than that obtained with the corticosteroid, betamethasone or the cyclooxygenase inhibitor, indomethacin. These results suggest that *in vivo* that PDE IV isozymes also play an important role in regulating the activity of T-cell mediated responses. However, since milrinone, SKF 94836 and zaprinast caused small but significant inhibitions, there seems to be some role for PDE III and PDE V isozymes. However, the inhibitory effect of SKF 94836 could be attributed to vascular vasodilatation, whilst that observed with milrinone (30%) may be accounted for by the fact that this compound also has an inhibitory effect on PDE IV isozymes (unpublished observations). The effect of zaprinast could be attributed to the inhibition of mediator release from mast cells,[14] one of the effector cells in the CS response known to contain PDE V isozymes. Nevertheless, since only partial inhibition was achieved by PDE IV inhibitors, there are likely to be other second messenger pathways involved in T-cell activation,[15] or alternatively only certain subsets of lymphocytes are sensitive to changes in intracellular cAMP levels.[3,13,16] The fact that the CS response can be inhibited to a greater extent by indomethacin and almost completely by betamethasone lends further support to these two suggestions. These observations taken together and the fact that PDE IV inhibitors are more potent at inhibiting the latter phase of the CS response suggests that these agents are acting mainly at the level of T cells.

In conclusion, PDE IV isozymes over and above other PDE isozyme subtypes, appear to play a major role in mediating CS responses. Since these PDE IV inhibitors in general are relatively weak inhibitors of the CS response and of the mitogen-induced T-cell proliferative response, these agents are likely to have poor immunosuppressant properties. In contrast, selective PDE IV inhibitors have been shown to have potent antiinflammatory effects in models of asthma in guinea-pigs[17] and inhibit IgE synthesis from human cells.[18] Therefore, these agents are likely to have greater potential as antiasthmatic agents but without the immunosuppressive effects of corticosteroids.

### References

1. Bos JD, Kapsenburg ML, Sillevis-Smitt JH. Pathogenesis of atopic eczema. *Lancet* 1994; **343**: 1388–1341.
2. Valette L, Prigent AF, Némoz G, Anker G, Macovschi O, Laggarde M. Concanavalin A stimulates the rolipram-sensitive isoforms of cyclic nucleotide phosphodiesterase in rat thymic lymphocytes. *Biochem Biophys Res Commun* 1990; **169**: 864–872.

3. Rosenthal LA, Taub DD, Moors MA, Blank KJ. Methylxanthine-induced inhibition of the antigen- and superantigen-specific activation of T and B lymphocytes. *Immunopharmacol* 1992; **24**: 203–217.
4. Li S-H, Chan SC, Toshitani A, Leung DYM, Hanifin JM. Synergistic effects of interleukin 4 and interferon-gamma on monocyte phosphodiesterase activity. *J Invest Dermatol* 1992; **99**: 65–67.
5. Thompson WJ. Cyclic nucleotide phosphodiesterases: pharmacology, biochemistry and function. *Pharmacol Ther* 1991; **51**: 13–33.
6. Marcoz P, Prigent AF, Lagarde M, Némoz G. Modulation of rat thymocyte proliferation response through the inhibition of different cyclic nucleotide phosphodiesterase isoforms by means of selective inhibitors and cGMP elevating agents. *Mol Pharmacol* 1993; **44**: 1027–1035.
7. Robicsek SA, Blanchard DK, Dje JY, Krzanowsk JJ, Szentivanyi A, Polson JB. Multiple high-affinity cAMP-phosphodiesterases in human T-lymphocytes. *Biochem Pharmacol* 1991; **42**: 869–877.
8. Mossman TR, Coffman RL. Different patterns of lymphokine secretion lead to different functional properties. *Ann Rev Immunol* 1989; **7**: 143–173.
9. Gocinski BL, Tigelaar RE. Roles of CD4+ and CD+ T cells in murine contact sensitivity revealed by *in vivo* monoclonal antibody depletion. *J Immunol* 1990; **144**: 4121–4128.
10. Kapsenberg ML, Wirenga EA, Bos JD, Jansen HM. Functional subsets of allergen-reactive human CD4+ T cells. *Immunol Today* 1991; **12**: 392–395.
11. Friedsman PS. Contact hypersensitivity. *Current Opinion Immunol* 1989; **1**: 690–693.
12. Cher DJ, Mossman TR. Two types of murine helper T cell clone. II. Delayed-type hypersensitivity is mediated by Th1 clones. *J Immunol* 1987; **11**: 3688–3694.
13. Krause DS, Deutsch C. Cyclic AMP directly inhibits IL-2 receptor expression in human T cells. *J Immunol* 1991; **146**: 2285–2294.
14. Fossard Y, Landry G, Pauli G, Ruckstahl M. Effects of cyclic AMP- and cyclic GMP-phosphodiesterase inhibitors on immunological release of histamine and on lung contraction. *Br J Pharmacol* 1981; **73**: 933–938.
15. Tamir A, Isakov N. Cyclic AMP inhibits phosphatidylinositol-coupled and -uncoupled mitogenic signals in T lymphocytes. *J Immunol* 1994; **152**: 3391–3399.
16. Munos E, Zubiaga AM, Merrow M, Sauter NP, Huber BT, Cholera toxin discriminates between T helper 1 and 2 cells in T cell receptor-mediated activation: role of cAMP in T cell proliferation. *J Exp Med* 1990; **172**: 90–103.
17. Lagente V, Moodley I, Perrin S, Mottin G, Junien JL. Effects of isozyme-selective phosphodiesterase inhibitors on eosinophil infiltration in the guinea-pig lung. *Eur J Pharmacol* 1994; **255**: 253–256.
18. Cooper KD, Kang K, Chan SC, Hanifin JM. Phosphodiesterase inhibition by Ro 20-1724 reduces hyper-IgE synthesis by atopic dermatitis cells *in vitro*. *J Invest Dermatol* 1985; **84**: 477–482.

**Received 26 September 1994; accepted in revised form 7 December 1994**
